# Genome sequences of six cluster CT and two cluster DJ bacteriophages that infect *Gordonia rubripertincta*

**DOI:** 10.1128/MRA.00736-23

**Published:** 2023-10-31

**Authors:** Xavier F. Rodriguez, Daniel C. Williams, Catherine P. Chia

**Affiliations:** 1 School of Biological Sciences, University of Nebraska-Lincoln, Lincoln, Nebraska, USA; 2 Department of Biology, Coastal Carolina University, Conway, South Carolina, USA; Department of Biology, Queens College, Queens, New York, USA

**Keywords:** actinobacteriophages

## Abstract

We report the genome sequences of eight bacteriophages isolated using *Gordonia rubripertincta* NRRL B-16540-SEA. Based on gene content similarity to phages in the Actinobacteriophage database, six of the phages are assigned to phage cluster CT while two are assigned to cluster DJ.

## ANNOUNCEMENT

The Science Education Alliance Phage Hunters Advancing Genomics and Evolutionary Science (SEA-PHAGES) project studies the genetic diversity and evolution of actinobacteriophages (1). Here, we report the identification and characterization of eight phages isolated using *Gordonia rubripertincta* NRRL B-16540-SEA, using standard methods (2). Briefly, soil samples collected from various sites in Omaha and Lincoln, NE, and Conway, SC (Table 1) were each washed with peptone-yeast extract-calcium medium, and the wash was filtered through a 0.22 µm filter. Filtrates were plated as top agar overlays with *G. rubripertincta* either immediately (direct isolation) or after inoculation with *G. rubripertincta* and incubated with shaking at 30°C for 2 days (enriched isolation). The direct isolation samples yielded phages BigChungus, Chikenjars, Dre3, Feastonyeet, and Gibbous, while enriched samples yielded phages Mayweather, Nithya, and Vine. All phages were plaque-purified through multiple rounds of plating. Negative-stain transmission electron microscopy revealed a siphovirus morphology for the phages (Mayweather was not imaged).

**TABLE 1:** Characteristics of genomes of eight phages isolated using host *G. rubripertincta* NRRL B-16540 SEA

Phage (cluster; year isolated)	GPS coordinates of soil collection sites	No. of sequencing reads	Approximate shotgun coverage (X)	Genome length (bp)	GC content (%)	3′ Overhang	No. of CDS^*a*^	CDS assigned function
BigChungus (CT; 2019)	41.2097 N, 96.2161 W	575,645	1724	47,166	60.7	CGGCGGTAGGCTT	69	33
Dre3 (CT; 2018)	40.8228 N, 96.6958 W	111,427	383	45,810	60.5	CGGTAGGCAT	68	33
Feastonyeet (CT; 2019)	41.2097 N, 96.2161 W	439,254	1310	47,166	60.7	CGGCGGTAGGCTT	69	33
Gibbous (CT; 2018)	40.8228 N, 96.6958 W	253,287	782	45,810	60.5	CGGTAGGCAT	68	33
Mayweather (CT; 2018)	33.903299 N, 79.062125 W	320,874	937	48,382	60.6	CGGCGGTAGGCTT	74	35
Vine (CT; 2020)	40.8203 N, 96.6967 W	67,933	202	48,092	60.4	CGGTAGGCTT	74	35
Chikenjars (DJ; 2018)	40.828025 N, 96.693583 W	320,431	735	61,544	51.3	CGCCGCTCT	92	20
Nithya (DJ; 2020)	40.8195 N, 96.6845 W	159,586	374	61,092	51.3	CGCCGCTCT	91	27

^*a*^ CDS, coding DNA sequences.

Genomic DNA was extracted from lysates with the Wizard DNA Clean-Up System (Promega), prepared for sequencing using the NEB Ultra II Library kit, and sequenced using an Illumina MiSeq (v3 reagents) to yield 150 bp single-end reads with indicated coverage (Table 1). Reads were assembled and checked for completeness using Newbler v2.9 and Consed v29.0, respectively (3 – 5). Table 1 provides genome lengths, GC%, and genome termini, determined by comparison to similar phages with known ends and confirmed by read start buildups (5). Based on gene content similarity of at least 35% to phages in the Actinobacteriophage database, phages were assigned to either phage cluster CT or DJ (6). Cluster CT phages BigChungus and Feastonyeet, which were isolated from different soil samples collected in the same location, differ only by 2 bp. Dre3 and Gibbous, similarly isolated, also differ by 2 bp.

Annotation of phage genomes was accomplished with DNA Master (7) (Version 5.23.3 or later), NCBI BLASTp (8), HHPred (9), GeneMark v2.5p (10), Glimmer v3.02 (11), Phamerator (12), SOSUI v1.11 (13), TMHMM v2.0 (14), and databases PhagesdDB (15) and Protein Data Bank (16). All software programs were run using default parameters.

For all six cluster CT phages, predicted L-Ala-D-Glu peptidase and glycosyl hydrolase domains of lysin A, often a single multi-domain protein in actinobacteriophages, are encoded by separate but adjacent genes. These are located on the left arm of the genomes and transcribed rightward. A predicted lysin B is encoded among DNA metabolism genes on the right arm of the genome where most genes are transcribed leftward. The predicted tail assembly chaperone proteins were annotated with a (–1) translational frameshift conserved in mycobacteriophage (17, 18). For the Cluster DJ phages, functional assignments were made for 20 of 92 genes for Chikenjars and 27 of 91 genes for Nithya. These include two major tail proteins, and a major capsid and maturation protease fusion protein. None of the eight phages encode identifiable tRNA genes.

## Data Availability

Under BioProject PRJNA488469, the following are the sequence reads in the sequence read archive (SRA) and Accession numbers: BigChungus (SRX11422988; GenBank MT776810), Chikenjars (SRX11422995; GenBank MN204501), Dre3 (SRX11422996; GenBank MW507135), Feastonyeet (SRX11422997; GenBank MT776808), Gibbous (SRX11422998; GenBank MN310549), Mayweather (SRX11555142; GenBank MN062716) Nithya (SRX11422991; GenBank MZ388556) and Vine (SRX11422992; GenBank MZ622167).
